# Prevalence of *Helicobacter pylori* infection and effectiveness of first-line triple eradication therapy among dyspeptic patients at hospitals in Hawassa City, Ethiopia: a cross-sectional follow-up study

**DOI:** 10.1186/s13099-024-00618-8

**Published:** 2024-04-27

**Authors:** Sintayehu Fekadu, Seyife Kibru, Sisay Tesfaye, Tariku Egeno, Alemu Tamiso, Hizkel Engiso, Serawit Deyno

**Affiliations:** 1 School of Laboratory Sciences, College of Medicine and Health Sciences, Hawassa University, P. O. Box 1560, Hawassa, Ethiopia; 2 Department of Internal Medicine, College of Medicine and Health Sciences, Hawassa University, P. O. Box 1560, Hawassa, Ethiopia; 3 Internal Medicine and Cardiology Unit, Abem Primary Hospital, P. O. Box 1162, Hawassa, Ethiopia; 4 School of Public Health, College of Medicine and Health Sciences, Hawassa University, P.O. Box 1560, Hawassa, Ethiopia; 5 School of Pharmacy, College of Medicine and Health Sciences, Hawassa University, P. O. Box 1560, Hawassa, Ethiopia

**Keywords:** Dyspepsia, *H. Pylori*, Eradication therapy, Fecal-antigen test, Hawassa

## Abstract

**Background:**

Dyspepsia is a common gastrointestinal illness sometimes associated with *Helicobacter pylori* (*H. pylori*) infection. Screening and eradicating the bacterium reduces the risk of infection-related complications. The aim of this study was to determine the magnitude of *H. pylori* infection among dyspeptic patients and the effectiveness of triple eradication therapy at hospitals in Hawassa city, Ethiopia.

**Results:**

The prevalence of *H. pylori* infection was 48.5%. The *H. pylori* eradication rate using first-line triple therapy was 83.8%. Eradication therapy failure is associated with previous exposure compared to no exposure (AOR: 4.8, 95% CI: 1.37–10.97), a regimen for 10-days compared to 14-days (AOR: 4.05, 95% CI: 1.42–11.55), and self-reported side effects compared to no report (AOR: 2.5, 95% CI: 1.12–5.97). Based on Morisky-eight scale 230 (79.0%) patients were adherent to their triple therapy. Participants with no reports of adverse effects showed increased odds of adherence to triple therapy compared to those who had reports (AOR = 2.45, 95% CI: 1.29–4.62).

**Conclusions:**

This study demonstrated that about half of adult dyspeptic patients were infected with *H. pylori*, and moderate eradication was observed. Factors such as previous history of eradication therapy, duration of the eradication regimen, and perception of potential adverse effects are associated with eradication rate and should be considered during the initiation of eradication therapy.

## Background

Dyspepsia is a complex disorder with several distinct pathophysiologic mechanisms. 70% of cases of dyspepsia are classified as functional dyspepsia (FD), which is characterized as epigastric discomfort (postprandial fullness, early satiety, and burning) for at least one month without any organic disease evidence discovered during upper endoscopy [1]. The global prevalence of FD was 20.8%, and it ranges from 7 to 45% depending on the dyspepsia definition used, geographical location, environmental risk factors, lifestyle, and socioeconomic status [2, 3]. Among patients with functional gastrointestinal disorders, the magnitude of FD was found to be 48.4% according to the Rome III criteria in Ethiopia [4]. Untreated dyspepsia is associated with patients’ poor quality of life, such as anxiety, depression and somatization [5].

*Helicobacter pylori* (*H. pylori*) infection is a well-known risk factor for gastrointestinal disorders, including dyspepsia. It has several virulent factors that promote its survival and cause a range of clinical conditions, such as gastritis, peptic ulcer, gastric carcinoma, and mucosa-associated lymphoid tissue (MALT) lymphoma. The three main pathogenic mechanisms associated with *H. pylori* virulence factors are immune evasion, disease induction and colonization. The virulence factor that causes colonization are adhesins, flagella, urease and the chemotaxis system. *H. pylori* are able to remain in the human stomach by evading the host immune clearance through proteins that are responsible for immune escape. Vacuolating-cytotoxin A (vacA) and cytotoxin-associated gene A (cagA) are responsible for direct damage of infected gastric epithelial cells and development of clinical diseases [6, 7]. The infection is acquired in early childhood via family close contact or poor sanitary conditions [8]. A recent systematic review and meta-analysis showed that the global *H. pylori* pooled prevalence ranges from 24% to70%, being the highest in the developing countries for low socioeconomic reasons and underdevelopment [9–12]. The prevalence of *H. pylori* infection in Ethiopia was 52.2% [13].

Eradication of *H. pylori* is the first-line treatment for infected patients with dyspepsia symptoms, as it can reduce symptoms in the majority of them and minimize the risk of serious complications, including the development of gastric cancer [14]. Different regimens have been used as first-line eradication therapy, such as clarithromycin-based triple therapies, bismuth-free therapies or bismuth-based quadruple therapies (BQT) [15, 16]. The choice of treatment protocol depends on the best local practice, antibacterial resistance patterns, cost, and availability of the drugs. The relative effectiveness of varying combinations of eradication regimens is affected by several factors, and the choice of antibiotics should be localized [17]. In general, as a first-line treatment, 14-days concomitant therapy, or 14-days BQT, is recommended in areas of high clarithromycin resistance (≥ 15%), whereas 14-days triple therapy, or 14-days BQT is recommended in areas of low clarithromycin resistance (< 15%) [18]. The success or failure of treatment is usually determined after a month of completion of the regimen using *H. pylori* detection techniques such as urea breath test, fecal antigen testing (FAT), or biopsy-based testing [19]. The FAT for *H. pylori* had good performance and was cost-effective for screening as well as confirmation of eradication [20].

The *H. pylori* drug resistance is increasing globally and has resulted in the failure of both first-line and second-line eradication therapy, which urges local surveillance network to select an appropriate eradication regimen [21]. There are a few studies in Ethiopia regarding *H. pylori* drug resistance. The trends of resistance against commonly used antimicrobial metronidazole was 5.26% (in 1999), 76% (in 2004), and 91.6% (in 2023); clarithromycin was 0% (in 2004), and 66.7% (in 2023); amoxicillin was 6% (in 2004), and 91.6% (in 2023); tetracycline was 0% (in 2004), and 37.5% (in 2023) [22–24]. This condition is significant, especially for the clarithromycin-based triple therapy. The key antibiotic clarithromycin becomes ineffective because of prior exposure to or use of other macrolides for treatment of infections [25]. Based on the status of clarithromycin resistance, regimens are optimized to maximize eradication rate. Moreover, evaluation of the current regimens and implementing evidence-guided eradication therapy improve the overall eradication rate and cure.

Very few studies are available on the effectiveness of *H. pylori* first-line eradication therapy among dyspeptic patients in Ethiopia. In addition, the recurrence rate of dyspepsia after taking eradication therapy is very common. The present study is significant because it addresses the public health issue on the effectiveness of *H. pylori* eradication therapy. Given the different regimens used to eradicate *H. pylori* infection, an assessment of the relative effectiveness of each regimen is critical for optimizing treatments and revision of protocols. The study benefits those patients whose eradication failed using the first-line eradication regimen to seek the second-line eradication regimen. Moreover, this study will contribute to the existing literature by providing factors that may affect *H. pylori* eradication.

Therefore, the aim of this study was to determine the prevalence of *H. pylori* infection and the effectiveness of eradication therapy among adult dyspeptic patients visiting gastroenterology outpatient departments in Hospitals in Hawassa city, Ethiopia.

## Methods

### Sampling, questionnaires, and dyspepsia screening

**Fig. 1:**
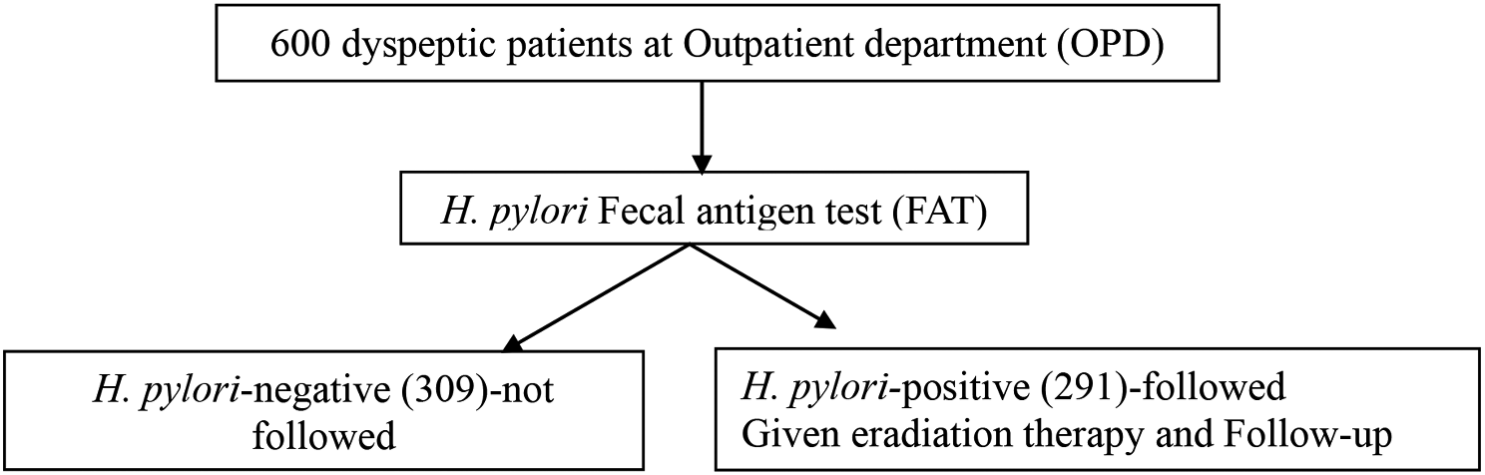
Recruitment of dyspeptic patients and selection of *H. pylori-*positive participants for follow-up of eradication therapy

The study was conducted in Hospitals in Hawassa city. Hawassa is the capital city of the Sidama region of Ethiopia. The study participants were recruited from five hospitals: four government hospitals (Hawassa comprehensive and specialized, Adare general, Motite Fura primary, and Millenium primary), and one private hospital (Abem primary hospital of internal medicine and cardiology unit). Most of the participants were from Hawassa and environs, for improved follow-up. A facility-based cross-sectional follow-up study was conducted among adult (age ≥ 18 years) dyspeptic patients who visited the gastroenterology outpatient departments from February to June 2023. The sample size to determine the prevalence of *H. pylori* infection among dyspeptic patients was calculated by considering a previous study done in Ethiopia, which was 51.4% [26]. Using the single population proportion and a margin of error of 4%, 600 participants were invited to participate in the study. Out of the 600 study participants, 309 were *H. pylori*-negative on *H. pylori* fecal antigen test and excluded from the follow-up. For the follow-up study, a total of 291 *H. pylori-*positive patients were followed to investigate the effectiveness of eradication therapy and drug adherence based on the previous study [27] and sample size calculation for the cohort study [28]. After getting their full consent, convenience sampling techniques were employed to invite them. The details of the recruitment of the study participants are presented in Fig. 1.

Study subjects were patients with functional dyspepsia who experienced the upper gastrointestinal disorder with the chief complaint of pain or burning in the stomach, bloating, excessive belching, or nausea after a meal and visited the outpatient department (OPD) during the study period. Patient selection and screening were performed by an experienced gastroenterologist or internist at the baseline. Data regarding the sociodemographic and clinical variables were recorded using structured questionnaires.

### FAT for *H. Pylori* infection and eradication confirmation

Stool samples were collected in a sterile plastic container for *H. pylori* FAT (SD BIOLINE *H. pylori* Ag, Standard Diagnostics, Inc. Korea). *H. pylori-*positive participants were given eradication therapy consisting of proton pump inhibitor (PPI)-based triple therapy: omeprazole 40 mg or pantoprazole 40 mg, twice a day for 15 to 30 days; clarithromycin (500 mg); and amoxicillin (1000 mg), each twice a day for 14-days or 10-days. After 4 weeks of completion of the eradication therapy, stool samples were collected and assayed as previously to confirm the success or failure of eradication. Self-reported information was used to estimate the adherence level. Medication adherence was measured by the Morisky Medication Adherence Scale–8 (MMAS-8), a measure of compliance with medication using time, dose, and frequency recommended by the health care provider [29]. A patient who scored seven no answers for the eight MMAS-8 was said to be adherent to triple therapy, while a patient who scored ≥ 2 yes answers for the MMAS-8 was said to be non-adherent to triple therapy.

### Statistical analysis

The data were entered, cleaned, and analyzed using SPSS version 25.0 (IBM Cop., Armonk, NY, USA). Descriptive statistics such as percentages, means, and standard deviations were used to describe the data. A Chi-square test was used to assess success-failure differences in *H. pylori* eradication. Bivariate and multivariable logistic regressions were used as predictors of failure of eradication and adherence rate. A backward stepwise logistic regression model was used during multivariable logistic regression to control confounding effect. The odds ratio with 95% confidence intervals was calculated for each of the independent variables using p-value < 0.05 as the level of significance.

## Results

Among 600 adult dyspeptic patients who had clinical signs and symptoms of dyspepsia, 291 were positive for *H. pylori* FAT giving an overall prevalence of 48.5% (291/600). The 291 *H. pylori-* positive patients were further consented to for follow-up. Among the 291 consented for the follow-up, 219 were able to came to hospitals to confirm their *H. pylori* eradication after one month of completion of the eradication therapy, and for the rest of the participants, their *H. pylori* status was confirmed at their home because of their social condition. The mean age of study subjects was 33.6 (± 12.04) years (range = 18–78 years), with most (38.8%) being between 25 and 34 years of age. Most of the participants were females (65.5%), resulting an overall female-to-male ratio of 1.8:1. Most of the participants live in Hawassa, and the remaining came from the nearby administrative districts. Tables 1 and 2 show the general physical and sociodemographic characteristics of the study participants.

**Table 1:** General physical characteristics of dyspeptic patients attending selected hospitals in Hawassa city administration, February–June, 2023

Variable	Participant (*n*)	Minimum	Maximum	Mean	SD
Age (years)	600	18	78	33.6	12.042
Height (m)	600	1.5	1.86	1.65	0.069
Weight before therapy (Kg)	600	40	95	58.277	9.910
Weight after therapy (Kg)	291	40	92	58.31	9.828
BMI before therapy	600	13.84	31.63	21.35	3.160
BMI after therapy	291	14.17	31.25	21.38	3.120

**Table 2:** Sociodemographic characteristics of dyspeptic patients attending selected hospitals in Hawassa city administration, February–June, 2023

Variable	Category	Frequency (%)
Sex	Male	207 (34.5)
	Female	393 (65.5)
Age (Years)	18–24	160 (26.7)
	25–34	233 (38.8)
	35–44	112 (18.7%)
	45–54	56 (9.3%)
	> 55	39 (6.5%)
Residence	Urban	375 (62.5%)
	Rural	225 (37.5%)
Marital status	Single	203 (33.8%)
	Married	376 (62.7%)
	Divorced	14 (2.3%)
	Widowed	7 (1.2%)
Religion	Orthodox Christian	397 (66.2%)
	Protestant	165 (27.5%)
	Muslim	32 (5.3%)
	Others	6 (1%)
Occupation	Housewife	123 (20.5)
	Government employee	119 (19.8)
	Private employee	102 (17.0)
	Farmer	97 (16.2)
	Student	35 (5.8)
	Others	117 (19.5)
Educational status	Illiterate	75 (12.5)
	Primary school	62 (10.3)
	Secondary school	157 (26.2)
	College and above	306 (51.0)
Income level (Eth birr)	No report	313 (52.2)
	< 2000	54 (9.0)
	2000–5000	111 (18.5)
	> 5000	122 (20.3)

### Medical information and *H. pylori* prevalence

The study participants were assessed for their medical conditions and tested for *H. pylori* status and eradication rate after completion of the eradication regimen. Among the subjects, 54.3% had a previous history of gastritis, 12.7% had a history of *H. pylori* infection, and 36.5% had a history of other illnesses. The clinical characteristics and associated factors are shown in Table 3.

**Table 3:** Clinical characteristics of dyspeptic patients attending selected hospitals in Hawassa city administration, February–June, 2023

Variable	Category	Frequency (%)
History of gastritis/PUD	Yes	326 (54.3)
	No	274 (45.7)
History of *H. pylori* infection	Not known	302 (50.3)
	Positive	76 (12.7)
	Negative	222 (37.0)
History of *H. pylori* eradication	Yes	38 (50)
	No	38 (50)
Duration of illness	Less than a month	188 (31.3)
	More than a month	412 (68.7)
Timing of dyspepsia feeling	Long interval between meals	44 (7.3)
	Before meal	106 (17.7)
	After meal	288 (48.0)
	Always	132 (22.0)
	At night	30 (5.0)
History of other diseases	Yes	219 (36.5)
	No	381 (63.5)
History of alcohol drinking	Yes	78 (13.0)
	No	522 (87.0)
History of traditional medicine use	Used	59 (9.8)
	Not used	541 (90.2)
*H. pylori* FAT	Positive	291 (48.5)
	Negative	309 (51.5)
Eradication regimen	OAC for 14-days	271 (93.1)
	OAC for 10-days	20 (6.9)
Self-reported after therapy	Improved	209 (34.8)
	Partially improved	40 (6.7)
	No improvement at all	38 (6.3)
	On worsening	4 (0.7)
Self-reported treatment completeness	Complete	219 (75.3)
	Mostly complete	41 (14.1)
	Partially complete	31 (10.6)
*H. pylori* eradication	Eradicated	244 (83.8)
	Not eradicated	47 (16.2)

### *H. Pylori* eradication and associated factors

Binary and multivariable logistic regression were performed to assess factors that are related to the *H. pylori* eradication rate among dyspeptic patients. Accordingly, after adjusting all the confounders, the previous history of *H. pylori* eradication and current eradication regimen used were associated with *H. pylori* eradication failure. Table 4 shows the binary and multivariable logistic regression of *H. pylori* eradication among the study participants.

**Table 4:** Binary and multivariable logistic regression of factors associated with *H. pylori* eradication in the hospitals in Hawassa city, February–June 2023 (*n* = 291)

Variable	Category	FAT after HPET		COR (CI: 95%)	*P*-value	AOR (CI: 95%)	*P*-value
		Positive	Negative				
Sex	Male	15	79	–			
	Female	32	165	1.02 (0.52,1.99)			
Age (years)	18–24	14	65				
	25–34	12	93	0.59 (0.26, 1.38)	0.23		
	35–44	8	44	0.84 (0.33, 2.18)	0.73		
	45–54	6	24	1.16 (0.4, 3.37)	0.78		
	> 54	7	18	1.81 (0.63, 5.14)	0.27		
Residence	Urban	16	146	–			
	Rural	31	98	2.89 (1.49, 5.56)	0.002	0.50 (0.22, 1.16)	0.106
Educational status	Illiterate	4	30	–			
	Primary school	7	25	2.1 (0.55, 8.01)	0.28		
	Secondary school	23	60	2.88 (0.91, 9.07)	0.07	1.88 (0.41, 8.71)	0.42
	College and above	13	129	0.76 (0.23, 2.48)	0.64		
History of gastritis	No	19	99	–			
	Yes	28	145	1.01(0.53, 1.90)	0.98		
History of *H. pylori* status	Not known	23	106	–			
	Positive	15	32	2.16 (1.01, 4.63)	0.04	1.21 (0.36, 4.02)	0.75
	Negative	9	106	0.39 (0.17, 0.89)	0.02	0.81 (0.31, 2.09)	0.65
History of eradication therapy	No	33	228	–			
	Yes	14	16	6.05 (2.70, 13.52)	0.001	4.82 (1.37, 16.97)	**0.014**
Duration of illness	Less than a month	12	80	–			
	More than a month	35	164	1.42 (0.70, 2.88)	0.33		
History of other illnesses	No	28	154	–			
	Yes	19	90	1.16 (0.61, 2.20)	0.65		
History of alcohol intake	No	8	38	–			
	Yes	39	206	0.89 (0.39, 2.07)	0.8		
History traditional medicine use	No	37	219	–			
	Yes	10	25	2.37 (1.05, 5.33)	0.037	1.74 (0.64, 4.71)	0.27
Eradication regimen used	OAC for 14-days	32	232	–			
	OAC for 10-days	8	12	3.96 (1.52, 10.32)	0.005	4.05 (1.42, 11.55)	0.009*
Regimen completeness	Complete	22	185	–			
	Incomplete	25	59	3.56 (1.87, 6.78)	0	1.34 (0.59, 2.99)	0.47
Adverse effect self-report	Present	36	107	–			
	Absent	11	137	4.19 (2.04, 8.62)	0	2.58 (1.12, 5.97)	0.026*
Adherence	Adhered	35	195				
	Not-adhered	12	49	0.73 (0.35, 1.52)	0.402	–	–

*OAC* Omeprazole Amoxicillin Clarithromycin, *COR* Crude Odds Ratio, *AOR* Adjusted Odds Ratio, *FAT* Fecal Antigen Test, *HPET H. pylori* Eradication Therapy

*Significant

### Adherence rate

Based on MMAS-8, 230 (79.0%) patients were adherent to their triple therapy medications, and 61 (21.0%) were non-adherent, Table 5.

**Table 5:** Morisky-Medication Adherence Predictor Scale-8 (MMAPS-8)

Characteristics	Response	Frequency	Percentage
Do you sometimes, forget to take your medications?	Yes	15	5.2
	No	256	94.8
Thinking over the past 2 weeks, were there any days when you did not take your medicine?	Yes	12	4.1
	No	279	95.9
Have you ever cut back or s topped taking your medication without telling your doctor, because you felt worse when you took it?	Yes	22	7.6
	No	269	92.4
When you travel or leave home, do you sometimes forget to bring along your medication?	Yes	34	11.7
	No	257	88.3
Did you forget to take your medicine yesterday?	Yes	4	1.4
	No	287	98.6
When you feel like your condition is under control, do you sometimes stop taking your medicine?	Yes	2	0.7
	No	289	99.3
Do you ever forget taking your medications properly as prescribed?	Yes	3	1
	No	288	99
How often do you forget taking your medication	Sometimes	23	7.9
	Not at all	268	92.1
Adherence to triple therapy medication	Adhered	230	79
	Not adhered	61	21

### Factors associated with adherence to triple therapy

The results of multivariate regression are indicated in Table 6. Except for the presence of self-reported adverse effects, no significant association with adherence was observed in both bivariate and multivariate models. Participants who have no reported adverse effects showed 1.80 times increased odds of adherence (COR = 1.80, 95% CI (1.01, 3.21**))** to triple therapy as compared to participants who has reported adverse effects. In the multivariate model, those who have no reports of adverse effects showed 2.45 times (AOR = 2.45, 95% CI (1.29–4.62) increased odds of adherence to triple therapy compared to those who have reports of adverse effects, Table 6.

**Table 6:** Binary and multivariable logistic regression of factors associated with adherence to *H. pylori* eradication therapy in the hospitals in Hawassa city, February–June 2023 (*n* = 291)

Variable	Category	Adherence		COR (CI: 95%)	*P*-value	AOR (CI: 95%)	*P*-value
		Adhered	Not-adhered				
Sex	Male	77	17			–	–
	Female	153	44	1.30 (0.70, 2.43)	0.41	–	–
Age (years)	18–24	60	19	–	–	–	–
	25–34	86	19	0.60 (0.18, 1.97)	0.40	–	–
	35–44	41	11	0.86 (0.26, 2.80)	0.80	–	–
	45–54	22	8	0.71 (0.20, 2.50)	0.59	–	–
	> 54	21	4	0.52 (0.14, 2.00)	0.34	–	–
Residence	Rural	122	40	–	–	–	–
	Urban	108	21	1.69 (0.93, 3.03)	0.08	0.49 (0.23, 1.01)	0.52
Educational status	Illiterate	28	6	–	–	–	–
	Primary school	25	7	1.47 (0.56, 3.84)	0.43	0.77 (0.21, 2.85)	0.69
	Secondary school	69	14	1.12 (0.45, 2.83)	0.80	1.48 (0.48, 4.63)	0.50
	College and above	108	34	1.55 (0.78, 3.10)	0.21	0.85 (0.30, 2.48)	0.77
History of gastritis	No	94	24	–	–	–	–
	Yes	136	37	1.066 (0.60, 1.90)	0.83	–	–
History of *H. pylori* status	Not known	102	27	–	–	–	–
	Positive	40	7	1.16 (0.63, 2.12)	0.63	0.92 (0.46, 1.81)	0.83
	Negative	88	27	1.753 (0.70, 4.36)	0.23	1.79 (0.69, 4.69)	0.23
History of eradication therapy	No	205	56	–	–	–	–
	Yes	25	5	0.73 (0.27, 2.00)	0.54	–	–
Duration of illness	Less than a month	77	15	–	–	–	–
	More than a month	153	46	1.54 (0.81, 2.94)	0.19	0.54 (0.27, 1.08)	0.08
History of other illnesses	No	144	38	–	–	–	–
	Yes	86	23	1.01 (0.57, 1.81)	0.97	–	–
History of alcohol intake	No	37	9	–	–	–	–
	Yes	193	52	1.11 (0.50, 2.44)	0.80	–	–
History traditional medicine use	No	199	57	–	–	–	–
	Yes	31	44	0.45 (0.15, 1.33)	0.15	2.35 (0.74, 7.45)	0.15
Eradication regimen used	OAC for 14-days	217	54	–	–	–	–
	OAC for 10-days	13	7	2.16 (0.82, 5.68)	0.12	0.40 (0.14, 1.12)	0.08
Regimen completeness	Complete	163	44	–	–	–	–
	Incomplete	67	17	0.94 (0.50, 1.76)	0.85	–	–
Adverse effect self-report	Present	106	37	–	–	–	–
	Absent	124	24	1.80 (1.01, 3.21)	0.04	2.45 (1.29, 4.62)	0.006

## Discussions

The prevalence of *H. pylori* infection among adult dyspeptic patients in this study was 48.5%. A decrease in prevalence was, of course, not a prefect consistency, what was observed in the most recent study reports is comparable with our findings, 51.4% [26]; 51.1% [30]; 49% [31]; 52.4% [32]; 49.2% [33]. Two of the studies with lower findings, 37.6% [34] and 34% [35] than the current study was published in 2017 and 2023. This inconsistency could be due to geographical variation. The oldest reports (published 20-years ago) showed *H. pylori* prevalence of 81% [36], far from the prevalence of recent publications. The reasons for decrease rate of *H. pylori* infection with time may be due to improved socioeconomic and personal hygiene [37, 38]. Several other factors could also be in display, such as improved nutritional quality and medical care overtime.

*H. pylori* infection results in persistent chronic infection, and the presence of other risk factors can lead to severe gastrointestinal diseases such as gastritis, peptic ulcer, and gastric cancer. Therefore, early screening and eradicating the bacteria decrease infection-associated complications [39]. In this study, the *H. pylori* eradication rate using standard triple therapy was 83.8% (244/291). This result was lower than the eradication rate reported from Northern Ethiopia, which was 90.3% [27] and higher than the study reported from Central Ethiopia, which was 50.0% [40]. Moreover, our findings are comparable with a recent systematic review on eradication rate of African studies, which was 79.0% [41]. Several factors are associated with failure in the eradication rate, such as eradication regimen used, pre-antibiotic resistance, poor drug compliance, and sociodemographic factors [42–45]. Overall, this eradication rate is acceptable in areas of low clarithromycin resistance (< 15%), one of the core antibiotics for the treatment of *H. pylori.* However, there is no local data regarding clarithromycin resistance levels, making it unlikely that this regimen will be continued.

In our study, the odds of eradication failure were four times higher in participants with previous exposure to *H. pylori* eradication therapy compared with those without exposure. This is supported by similar studies conducted elsewhere showing the importance of considering the patient’s antibiotic history before employing first-line eradication therapy [46–48]. Exposure to antibiotics indeed accelerated antimicrobial resistance and, thus, treatment failure.

In multivariate logistic regression, patients receiving an eradication regimen consisting of omeprazole, amoxicillin, and clarithromycin for 10-days had four times more eradication failures than patients receiving the same regimen for 14-days. The effectiveness of eradication depends on the regimen and successful adherence to the treatment protocol. Several studies supported the finding that the 14-days treatment showed better eradication compared to the 10-days regimen [49–52]. However, another study showed no difference in eradication rate was observed in patients taking a 10-days and 14-days triple eradication regimen [53].

The effectiveness of *H. pylori* eradication depends not only on choosing the right antibiotic combination, but also on the proper taking of those antibiotics and proper counseling on perceived side effects. In our study, those participants who self-reported fewer or no side effects were two times more likely to experience eradication failure compared with those who had no self-reported adverse effects. Most of the eradication regimens have side effects, and adherence to the regimens during the treatment course is the main factor for success of eradication. The pretreatment consultation with physicians regarding the nature and effect of the antibiotics and follow-up during treatment also play a crucial role, as most patients become reluctant to take their medicine at home [54, 55].

The adherence rate and the eradication rate in this study showed similar values; however, the finding of the current study did not reveal an association. In this study, adherence to triple therapy was associated with reports of adverse effects. Better adherence was observed among those who did not report adverse effects. Adverse effects can compel patients to compromise treatment adherence and even force them to stop medications. In a previous study, 6.6% of patients on triple therapy with adverse events stopped medication [56]. The eradication rate of *H. pylori* on triple therapy is influenced by adherence in children [57]. The self-report modified Morisky adherence scale was used to assess medication adherence, which tends to overestimate adherence when compared to other methods such as pill counts, prescription claims or biological assays [58]. It also did not assess the outcome of adherence; future researchers are warranted to corroborate the finding.

The virulence factors, particularly the vacA and cagA status of the infecting *H. pylori* strain, are important for disease development and response to medical interventions such as eradication therapy. However, because of facility limitations in our study, the cagA and vacA statuses were not determined; hence, the samples were appropriately preserved and stored for future studies.

## Conclusions

This study demonstrated that about half of adult dyspeptic patients were infected with *H. pylori*, and this suggests careful consideration of dyspeptic patients for proper management. Our study also demonstrated a moderate *H. pylori* eradiation rate, 83.8% using first-line eradiation therapy, and this eradication rate is acceptable in areas of low clarithromycin resistance; however, so far, there is no published data in the study area to recommend that this regimen be continued for *H. pylori* eradication.

Careful assessment of dyspeptic patients regarding their previous history of eradication therapy, which may have resulted in poor eradication or pre-antibiotic exposure, ultimately leads to eradication failure. In addition, the selection of a longer duration over the shorter regimen, coupled with proper consultation during the course of treatment, promotes the rate of eradication.

## Data Availability

No datasets were generated or analysed during the current study.

## Abbreviations

BQT: Bismuth-based quadruple therapies
FD: Functional dyspepsia
FAT: Fecal antigen test
*H. pylori*: *Helicobacter pylori*
MMAS-8: Morisky Medication Adherence Scale-8
OPD: Outpatient department
PUD: Peptic ulcer disease
